# Retraction: Up-regulation of miR-34c-5p inhibits nasopharyngeal carcinoma cells by mediating NOTCH1

**DOI:** 10.1042/BSR-2020-0302_RET

**Published:** 2021-10-28

**Keywords:** MiR-34c-5p, NOTCH1

This article is being retracted from *Bioscience Reports* at the request of the Editor-in-Chief and the Editorial Board following receipt of a notification from a reader, alerting the Editorial Board to the backgrounds of the Western blots throughout the paper which unexplainedly contain the same features.

The authors have been contacted in regards to the Retraction, and have not responded to the Journal's queries and the concerns raised. Given the extent of the issues raised, the Editorial Board stand by the decision to retract the article.

